# Supplementary material for: Simulation of electroporation threshold based on the evolution of transmembrane potential and pore density
Source: PeerJ. 2025 Apr 28;13:e19356. doi: 10.7717/peerj.19356 (PMC12045266; doi:10.7717/peerj.19356)
Supplement: Supplemental Information 1 [file peerj-13-19356-s001.docx]

**Simulation of** **electroporation threshold based on the evolution of transmembrane potential and pore density**

**Changes in the electric field inside the cell before and after perforation.**

Figures S1(a)-(d) show the changes in the internal electric field of the cell before and after electroporation occurs after applying a 100 ns pulse and a 100 us pulse, respectively. Figures S1(a)-(b) apply an electric field strength of 4.8 kV, and S1(c)-(d) apply an electric field strength of 1.2 kV.

Figures S1 Changes in the electric field inside the cell. (a) t=0ns. (b) t=100ns. (c) t=0μs. (d) t=100μs.
